# Epidermal growth factor-induced cyclooxygenase-2 enhances head and neck squamous cell carcinoma metastasis through fibronectin up-regulation

**DOI:** 10.18632/oncotarget.2783

**Published:** 2014-12-22

**Authors:** Jinn-Yuan Hsu, Kwang-Yu Chang, Shang-Hung Chen, Chung-Ta Lee, Sheng-Tsung Chang, Hung-Chi Cheng, Wen-Chang Chang, Ben-Kuen Chen

**Affiliations:** ^1^ Institute of Basic Medical Sciences, College of Medicine, National Cheng Kung University, Tainan 701, Taiwan, ROC; ^2^ National Institute of Cancer Research, National Health Research Institutes and Division of Hematology/Oncology, Department of Internal Medicine, National Cheng Kung University, Tainan 701, Taiwan, ROC; ^3^ Division of Hematology and Oncology, Department of Internal Medicine, Chi-Mei Medical Center, Liouying, Tainan 736, Taiwan, ROC; ^4^ Department of Pathology, National Cheng Kung University Hospital, Tainan 701, Taiwan, ROC; ^5^ Department of Pathology, Chi-Mei Medical Center, Tainan 710, Taiwan, ROC; ^6^ Institute of Biochemistry, National Cheng Kung University, Tainan 701, Taiwan, ROC; ^7^ Graduate Institute of Medical Sciences, College of Medicine, Taipei Medical University, Taipei 110, Taiwan, ROC; ^8^ Department of Pharmacology, College of Medicine, National Cheng Kung University, Tainan 701, Taiwan, ROC; ^9^ Institute of Bioinformatics and Biosignal Transduction, College of Bioscience and Biotechnology, National Cheng Kung University, Tainan 701, Taiwan, ROC

**Keywords:** EGF, metastasis, COX-2, fibronectin

## Abstract

Epidermal growth factor receptor (EGFR) activation is a major cause of metastasis in many cancers, such as head and neck squamous cell carcinoma (HNSCC). However, whether the induction of cyclooxygenase-2 (COX-2) mediates EGF-enhanced HNSCC metastasis remains unclear. Interestingly, we found that EGF induced COX-2 expression mainly in HNSCC. The tumor cell transformation induced by EGF was repressed by COX-2 knockdown, and this repression was reversed by simultaneously treating the cells with EGF and prostaglandin E_2_ (PGE_2_). The down-regulation of COX-2 expression or inhibition of COX-2 activity significantly blocked EGF enhancement of cell migration and invasion, but the addition of PGE_2_ compensated for this inhibitory effect in COX-2-knockdown cells. COX-2 depletion inhibited EGF-induced matrix metalloproteinase (MMP)-1, MMP-2, MMP-3, MMP-9, and fibronectin expression and Rac1/cdc42 activation. The inhibitory effect of COX-2 depletion on MMPs and the fibronectin/Rac1/cdc42 axis were reversed by co-treatment with PGE_2_. Furthermore, depletion of fibronectin impeded the COX-2-enhanced binding of HNSCC cells to endothelial cells and tumor cells metastatic seeding of the lungs. These results demonstrate that EGF-induced COX-2 expression enhances HNSCC metastasis via activation of the fibronectin signaling pathway. The inhibition of COX-2 expression and activation may be a potential strategy for the treatment of EGFR-mediated HNSCC metastasis.

## INTRODUCTION

Head and neck squamous cell carcinoma (HNSCC) is the sixth most common cancer in the world [1]. Although there has been much research on the treatment of HNSCC, survival rates have improved little in the last 30 years [1, 2]. Over 50% of newly diagnosed patients do not achieve complete remission, and nearly 10% are recurrent cases with metastasis to distant organs [3]. Therefore, studies focusing on a deeper understanding of HNSCC to develop effective therapeutic strategies are required. Several biomarkers related to HNSCC pathogenesis and tumor progression have been described, including *TP53* mutations [4], the presence of human papillomavirus (HPV) [5] or its surrogate marker p16 [6] and altered expression of cyclooxygenase-2 (COX-2) and epidermal growth factor receptor (EGFR), which can provide prognostic information [1, 7, 8]. Cetuximab is currently the only EGFR-targeted drug approved for treating HNSCC. Cetuximab is used in combination with locoregional radiotherapy or chemotherapy in the recurrent and/or metastatic setting [9, 10]. However, the first-generation EGFR tyrosine-kinase inhibitors (TKIs) gefitinib and erlotinib show minimal tumor inhibition efficacy as monotherapies in HNSCC [11, 12]. Prostaglandin endoperoxide synthase, also known as COX-2, catalyzes the conversion of arachidonic acid to prostaglandins and thromboxanes [13, 14]. It is well known that the up-regulation of COX-2 contributes to increased antiapoptotic, angiogenic and metastatic potential in many types of cancer, such as lung, colon, breast, and pancreatic cancer and HNSCC cancers [15–17]. In addition, COX-2 is an early gene that is rapidly induced by pro-inflammatory cytokines (interleukin (IL) 1β, IL2 and tumor necrosis factor (TNF)), growth factors (EGF and platelet-derived growth factor (PDGF)), lipopolysaccharides, bile acids, ultraviolet B irradiation and tumor promoters [18–21]. In previous studies, COX-2 was found to be involved in cancer tumor cell metastasis by regulating biochemical changes, including altering matrix metalloproteinase (MMP)-2, MMP-9, and epithelial–mesenchymal transition (EMT) marker expression and increasing tumor cell adhesion to extracellular matrix (ECM) proteins and endothelial cells [22–24]. Interestingly, fibronectin is expressed in several types of carcinoma cells, and many studies have demonstrated a role for fibronectin in human solid tumor formation [25–27]; fibronectin can also regulate COX-2 expression [25, 28–30]. However, the function of fibronectin in COX-2-mediated metastasis remains unclear.

Similar to COX-2, EGFR is overexpressed in many human tumor types and is associated with poor prognosis and decreased survival [31]. Activation of the EGFR signaling pathway or expression of EGFR family members can impact tumor metastasis [32, 33]. EGFR activation leads to increased mitogen-activated protein kinase (MAPK) activity, resulting in aryl hydrocarbon receptor nuclear translocator (ARNT)/AP-1-mediated COX-2 expression [34, 35]. COX-2-derived prostaglandin E_2_ (PGE_2_) can activate EGFR signaling to stimulate cell proliferation. In addition, the correlation between COX-2 and the EGFR pathway in tumorigenesis has been demonstrated, suggesting that combination therapy with COX-2 and EGFR inhibitors would be more effective in tumor suppression than either agent alone [22, 36]. In clinical trials, dual functional blockade of EGFR and COX-2 in HNSCC and in lung cancer has been investigated [37, 38]. Notably, however, it is unknown whether COX-2 induction is correlated with EGF-enhanced HNSCC metastasis.

In this study, we reveal for the first time that the induction of COX-2 correlates with EGF-enhanced HNSCC metastasis. We demonstrate that EGF-induced COX-2 up-regulates the expression of MMP-1, MMP-2, MMP-3, MMP-9 and fibronectin and promotes the activation of Rac1/cdc42 to enhance HNSCC migration and invasion. These results indicate that EGF-induced COX-2 enhances HNSCC metastasis through the fibronectin/Rac1/cdc42 signaling pathway. COX-2 inhibition provides a new strategy for the treatment of EGFR-mediated HNSCC metastasis.

## RESULTS

### Induction of COX-2 expression and enhancement of anchorage-independent growth in EGF-treated HNSCC cells

**Figure 1:**
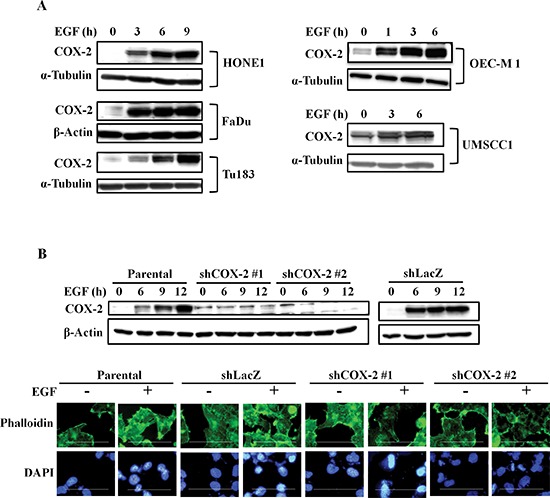
EGF induces COX-2 expression and morphological changes in HNSCC cells **(A)** HNSCC cells were treated with 50 ng/ml EGF in serum-free medium for the indicated amount of time. Cell lysates were prepared and subjected to SDS-PAGE and western blot analysis using antibodies against COX-2, α-tubulin and β-actin. **(B)** Upper panel, HONE1, shLacZ and shCOX-2 cells were treated with 50 ng/ml EGF in serum-free medium. Cell lysates were prepared, subjected to SDS-PAGE and analyzed by western blotting with antibodies against COX-2 and β-actin. Lower panel, HONE1 cells were treated with 50 ng/ml EGF in serum-free medium for 9 h and fixed with 4% paraformaldehyde, labeled with the f-actin-specific fluorescent dye, phalloidin. DNA was stained with 4′,6-diamidino-2-phenylindole (DAPI). Immunofluorescence images were captured using a microscope. Scale bar represents 100 μm.

We have previously reported that EGF induces COX-2 expression in A431 cells to enhance cell migration [19]. To further clarify whether the COX-2 induction is a general phenomenon of EGF-treated tumor cells, we examined several types of tumor cell lines. We found that EGF significantly induced COX-2 expression in various HNSCC cell lines (Figure 1A). However, the induction of COX-2 expression was not observed in other cell types, including breast cancer, lung cancer and colorectal carcinoma cells (Supplemental Figure S1A). We next investigated the association of the COX-2 gene expression signature with HNSCC by data mining using the cancer microarray database Oncomine 4.0 (Oncomine DB at http://www.oncomine.org) [39]. COX-2 expression in normal and malignant or metastatic tissues from HNSCC patients was compared using published datasets, and results demonstrated that COX-2 expression was higher in malignant tissues than in normal tissues from HNSCC patients (Supplemental Figure S1B). Significantly, COX-2 expression was remarkably higher in metastatic tissues (Supplemental Figure S1B). These results suggest that higher levels of COX-2 mRNA are expressed in clinical HNSCC tissues than normal tissues (*p* < 0.05). To study the role of EGF-induced COX-2 expression in HNSCC, we examined the effect of COX-2 on EGF-induced cellular transformation using soft agar assays. Lentiviral shRNA knockdown of COX-2 expression was performed in HONE1 cells (shCOX-2) (Figure 1B, upper panel), and results showed a decrease in EGF-enhanced actin polymerization following COX-2 silencing (Figure 1B, lower panel). Increases in size and number of colonies grown in soft agar in response to EGF stimulation were inhibited in shCOX-2 cells, whereas shCOX-2 cell colony formation in soft agar was restored by simultaneously treating cells with EGF and PGE_2_ (Supplemental Figures S2A and S2B). These results indicate that COX-2 contributes to EGF-induced cell transformation and cytoskeletal rearrangement.

### EGF-induced COX-2 regulates HNSCC migration and invasion

**Figure 2:**
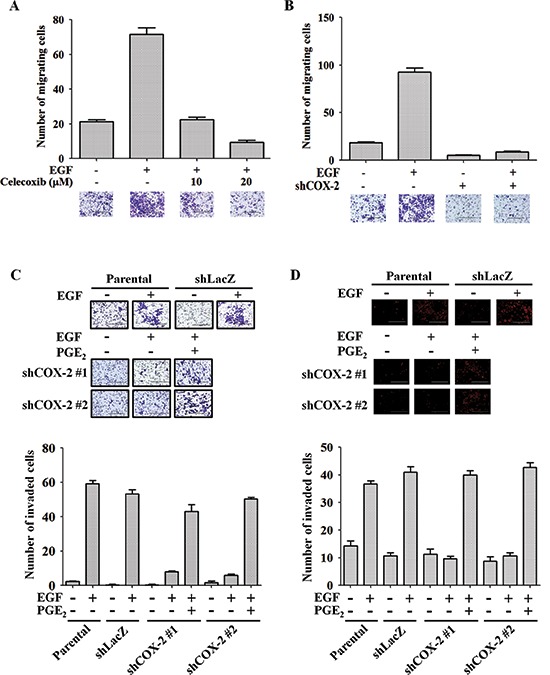
COX-2 regulates EGF-induced HNSCC cell migration and invasion **(A)** The migratory properties of HONE1 cells were analyzed using the transwell migration assay. Cells were treated with 50 ng/ml EGF and celecoxib in serum-free medium for 15 h. Upper panel, the number of migrating cells was determined under a microscope. Lower panel, representative photomicrographs. **(B)** HONE1 and shCOX-2 cells were treated with 50 ng/ml EGF in serum-free medium for 15 h. Upper panel, the number of migrating cells was determined using a microscope. Lower panel, representative photomicrographs. The number of migrating cells was determined using three randomly chosen fields under the microscope from three independent experiments. **(C)** The invasive properties of tumor cells were examined using the invasion assay as described in the “Materials and methods”. Parental and shCOX-2 (clone #1 and #2) HONE1 cells were treated with 50 ng/ml EGF and 10 μM PGE_2_ in serum-free medium for 48 h. Images were captured under a microscope (upper panel). The number of invaded cells was determined as shown in the lower panel. **(D)** The transendothelial invasion of tumor cells was performed as described in the “Materials and methods”. Parental and shCOX-2 (clone #1 and #2) HONE1 cells were treated with 50 ng/ml EGF and 10 μM PGE_2_ in serum-free medium for 48 h. Images of invaded cells were captured under a microscope (upper panel). The number of invaded cells was determined in three randomly chosen fields under a microscope in three independent experiments (lower panel). Scale bar represents 200 μm. Values represent means ± S.E.M.

EGFR activation is a major cause of metastasis in many cancers [33, 40]. COX-2 depletion also inhibited EGF-induced actin polymerization (Figure 1B, lower panel), indicating that COX-2 may participate in EGF-regulated cell mobility. To determine whether induction of COX-2 mediates EGF-induced tumor metastasis, we performed Transwell migration, invasion and transendothelial invasion assays. As shown in Figure 2A, inhibition of COX-2 activity with celecoxib dramatically reduced EGF-stimulated cell migration, suggesting that COX-2 activity is required for EGF-mediated cell migration. The inhibition of cell migration was further confirmed in shCOX-2 cells (Figure 2B). In addition, COX-2 depletion reduced EGF-stimulated cell invasion (Figure 2C), which was rescued when the shCOX-2 cells were simultaneously treated with PGE_2_ and EGF (Figure 2C). To further clarify whether EGF-induced COX-2 confers the ability to invade vessels in HNSCC cells, the transendothelial assay was utilized. EGF-induced transendothelial invasion was significantly reduced in COX-2 knockdown cells (Figure 2D and Supplemental Figure S3A); however, the simultaneous treatment of COX-2 knockdown cells with PGE_2_ and EGF restored their invasive capacity (Figure 2D and Supplemental Figure S3A). In addition, PGE_2_, the main product of COX-2 activity, enhanced transendothelial invasion and EGF-induced invasion (Supplemental Figure S3B). However, the EGFR inhibitor gefitinib blocked EGF-induced, but not PGE_2_-induced anchorage-independent growth and transendothelial invasion, indicating that PGE_2_ did not induce its effects on cellular function through the EGFR pathway (Supplemental Figures S4A and S4B). These results suggest that induction of COX-2 following EGFR activation is essential for EGF-induced cell invasion and transformation. In an *in vivo* metastasis assay, lung metastasis following tail vein injection of EGF-treated tumor cells was significantly increased in the parental but not the shCOX-2 cells (Supplemental Figure S5). These results indicate that EGF-induced COX-2 protein expression is essential for HNSCC metastasis.

### EGF-induced COX-2 activates the fibronectin/FAK/Rac1/cdc42 signaling axis

**Figure 3:**
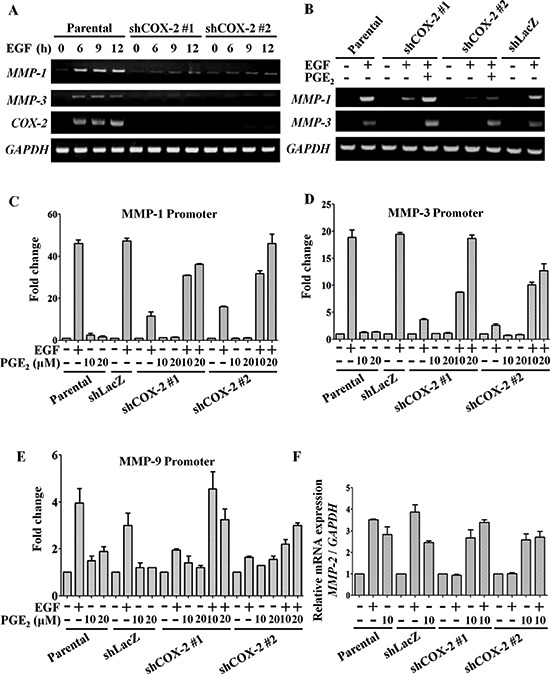
EGF-induced COX-2 enhances expression of MMP-1, MMP-2, MMP-3 and MMP-9 HONE1, shLacZ and shCOX-2 cells were treated with 10 μM PGE_2_ or 50 ng/ml EGF in serum-free medium for the indicated period of time. **(A, B)** Total RNA was extracted for reverse-transcription PCR with *MMP-1, MMP-3, COX-2* and *glyceraldehyde-3-phosphate dehydrogenase* (*GAPDH*) primers. **(C–E)** Cells were transfected with *MMP-1*, *MMP-3* and *MMP-9* promoters using lipofection. Cells were treated with 50 ng/ml EGF and 10 or 20 μM PGE_2_ in serum-free medium for 24 h. Luciferase activity and protein concentrations were then determined and normalized. Values represent means ± S.E.M. of three determinations. **(F)** Cells were treated with 50 ng/ml EGF or 10 μM PGE_2_ in serum-free medium for 9 h. The mRNA level of *MMP-2* was measured and normalized to *GAPDH* by real-time PCR. Values represent means ± S.E.M. of three determinations.

To clarify the mechanism involved in the regulation of tumor metastasis by EGF-induced COX-2, we examined changes in EMT markers in cells treated with EGF or PGE_2_. As shown in Supplemental Figures S6A and S6B, EGF significantly induced the expression of MMPs. To verify the role of COX-2 in the induction of MMP expression, the expression of MMP-1, MMP-3, MMP-9 and MMP-2 was examined in shCOX-2 cells. EGF-induced MMP-1 and MMP-3 expression was significantly inhibited in shCOX-2 cells (Figure 3A). Co-treatment with PGE_2_ restored the MMP-1 and MMP-3 levels in the EGF-treated shCOX-2 cells (Figure 3B). To further confirm whether the EGF-induced expression of MMP mRNA was via the induction of COX-2, the promoter activity of MMPs was examined in shCOX-2 cells. As shown in Figures 3C-3E, the EGF-induced promoter activity of MMP-1, MMP-3 and MMP-9 was inhibited in shCOX-2 cells. Although the down-regulation of MMP promoter activity in the EGF-treated shCOX-2 cells was reversed by PGE_2_ in a dose-dependent manner (Figures 3C-3E), MMP-1, MMP-3 and MMP-9 were not induced by PGE_2_ in the absence of EGF (Figures 3C-3E). However, both EGF and PGE_2_ significantly induced the expression of MMP-2 mRNA (Figure 3F and Supplemental Figure S6C). The inhibition of MMP-2 expression in EGF-treated shCOX-2 cells was also reversed by PGE_2_. These results reveal that the induction of COX-2 expression is essential for EGF-induced MMP-1, MMP-2, MMP-3 and MMP-9 expression. Nevertheless, activation of COX-2 affected MMP-2 expression but not MMP-1, MMP-3 or MMP-9, indicating that cooperation between COX-2 and EGF-activated signaling is required for MMP-1, MMP-3 and MMP-9 expression.

**Figure 4:**
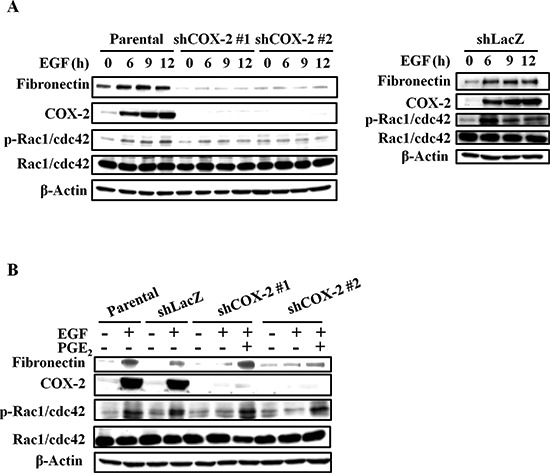
EGF-induced COX-2 enhances the expression of fibronectin and activation of Rac1/cdc42 **(A, B)** HONE1, shLacZ and shCOX-2 cells were treated with 50 ng/ml EGF and 10 μM PGE_2_ in serum-free medium for 9 h. Cell lysates were prepared, subjected to SDS-PAGE and analyzed by western blotting with antibodies against fibronectin, COX-2, Rac1/cdc42, phosphorylation of Rac1/cdc42 and β-actin.

**Figure 5:**
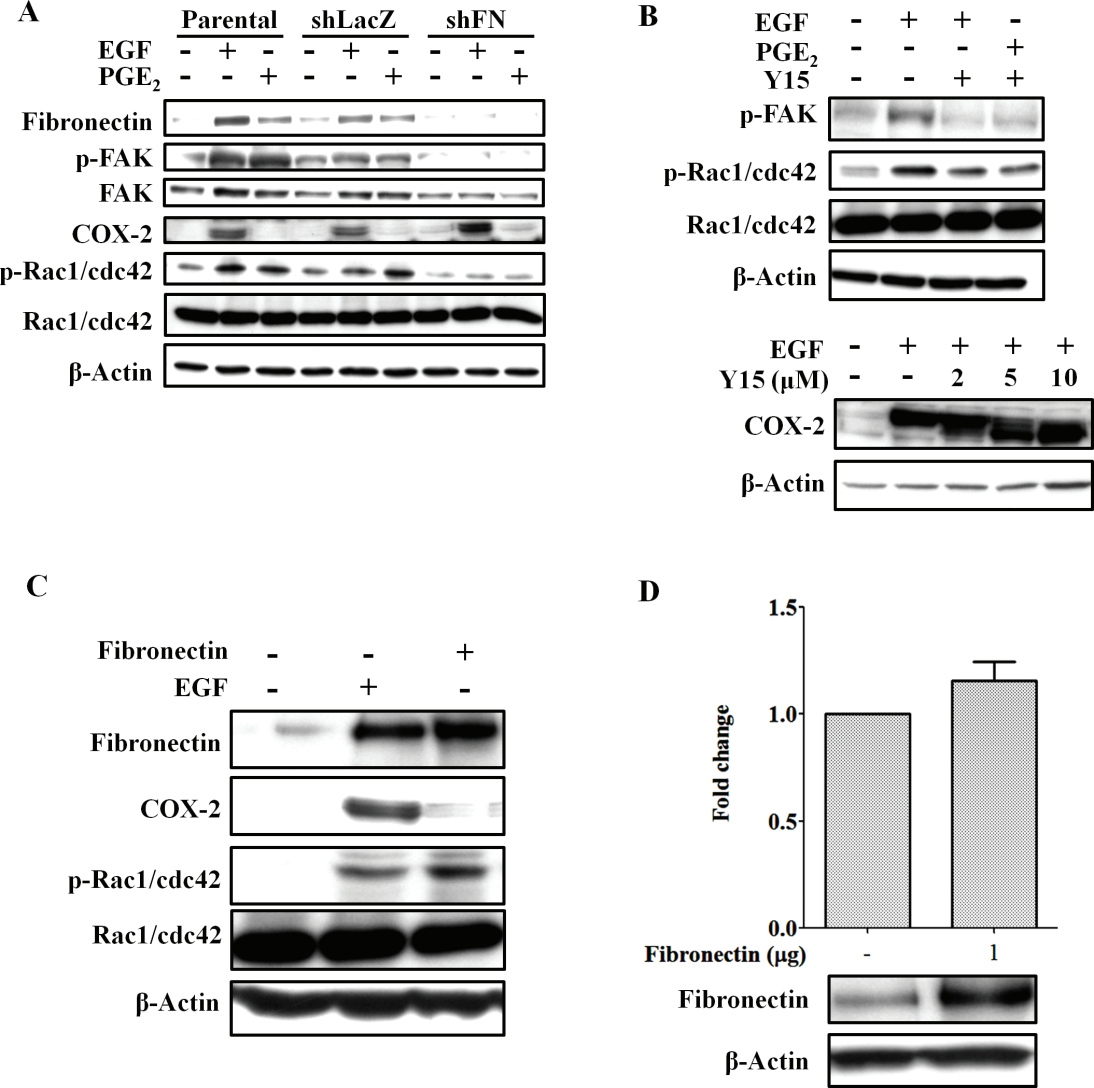
Knockdown of fibronectin inhibits EGF- and PGE_2_-induced activation of FAK and Rac1/cdc42 **(A)** HONE1, shLacZ and shFN (fibronectin knockdown) cells were treated with 50 ng/ml EGF and 10 μM PGE_2_ in serum-free medium for 9 h. Cell lysates were prepared, subjected to SDS-PAGE and analyzed by western blotting with antibodies against fibronectin, FAK, COX-2, β-actin, Rac1/cdc42, and phosphorylated FAK and Rac1/cdc42. **(B)** HONE1 cells were pretreated 10 μM FAK inhibitor 1,2,4,5-benzenetetraamine tetrahydrochloride (Y15) in serum-free medium for 1 h then treated with 50 ng/ml EGF and 10 μM PGE_2_ for 9 h. Cell lysates were prepared, subjected to SDS-PAGE and analyzed by western blotting with antibodies against COX-2, β-actin, Rac1/cdc42, and phosphorylated FAK and Rac1/cdc42. **(C)** HONE1 cells were treated with 50 ng/ml EGF in serum-free medium for 9 h or transfected with eGFP-fibronectin vector by lipofection. Cell lysates were prepared, subjected to SDS-PAGE and analyzed by western blotting with antibodies against fibronectin, COX-2, Rac1/cdc42, phosphorylated Rac1/cdc42 and β-actin. **(D)** HONE1 cells were transfected with eGFP-fibronectin and the COX-2 promoter by lipofection. Luciferase activity and protein concentrations were then determined and normalized. Values represent means ± S.E.M. of three determinations. Cell lysates were prepared, subjected to SDS-PAGE and analyzed by western blotting with antibodies against fibronectin, and β-actin.

EMT markers, such as slug, twist, vimentin, and N-cadherin were significantly induced by EGF (Supplemental Figure S7A). In addition, EGF also enhanced expression of fibronectin, phospho-Rac1/cdc42 and phospho-FAK (Supplemental Figures S7B and S7C). As shown above, fibronectin expression, activation of the FAK/Rac1/cdc42 axis downstream of fibronectin, and tumor migration were concomitantly induced by EGF. We examined whether EGF-induced changes in fibronectin and phospho-Rac1/cdc42, which in turn increase cell migration, are mediated by COX-2 induction in HNSCC. Although the EGF-induced expression of slug, twist, vimentin and N-cadherin was not altered in the shCOX-2 cells (data not shown), depletion of COX-2 dramatically inhibited EGF-induced expression of fibronectin (Figure 4 and Supplemental Figures S8A and S8B). Furthermore, the induction of phospho-Rac1/cdc42 expression by EGF was blocked in COX-2 knockdown cells (Figure 4A and Supplemental Figures S8A and S8B). The inhibition of fibronectin and phospho-Rac1/cdc42 in the COX-2 knockdown cells was overcome by PGE_2_ treatment (Figure 4B, and Supplemental Figures S8A and S8B). In addition, PGE_2_ alone also induced the expression of fibronectin and phosphorylation of Rac1/cdc42 in parental and COX-2 knockdown cells (Supplemental Figure S8). To further confirm that COX-2 activation was associated with cell mobility, the activation of phospho-FAK and phospho-Rac1/cdc42 was examined in cells treated with PGE_2_. As shown in Figure 5A, PGE_2_ significantly induced the expression of fibronectin and the activation of phospho-FAK/Rac1/cdc42, similar to the effects of EGF stimulation. Although EGF- and PGE_2_-induced phospho-FAK/Rac1/cdc42 signaling was inhibited in cells treated with compound Y15 (Figure 5B), which specifically inhibits FAK-Y397 autophosphorylation [41–43], no effect of Y15 on EGF-induced COX-2 expression was observed (Figure 5B). To further clarify the correlation between fibronectin and COX-2 expression, the effect of fibronectin overexpression or knockdown on COX-2 expression in EGF-treated cells was examined. As shown in Figures 5C and 5D, fibronectin overexpression activated phospho-Rac1/cdc42; however, fibronectin did not affect COX-2 expression. Lentiviral shRNA knockdown of fibronectin expression was performed in HONE1 cells (shFN). In addition, depletion of fibronectin inhibited phospho-FAK and phospho-Rac1/cdc42 but had no effect on EGF-induced COX-2 expression (Figure 5A and Supplemental Figures S9A and S9B). These results indicate that induction of the fibronectin/FAK/Rac1/cdc42 signaling pathway is dependent on EGF-induced COX-2 expression and activation.

### Depletion of fibronectin inhibits PGE_2_-induced tumor invasion and interaction with endothelial cells

**Figure 6:**
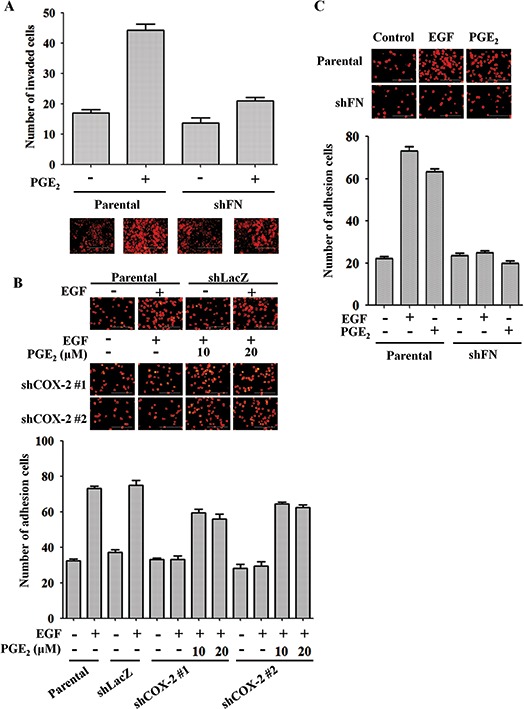
Fibronectin knockdown inhibits EGF- and PGE_2_-mediated induction of HNSCC invasion and adhesion to endothelial cells **(A)** The invasive properties of HONE1 and shFN cells were analyzed using transendothelial invasion assay. Cells were treated with 10 μM PGE_2_ in serum-free medium for 48 h. Upper panel, the number of invaded cells were determined under a microscope. Images of invaded cells were captured under a microscope (lower panel). The number of invaded cells was determined using three randomly chosen fields under a microscope from three independent experiments. **(B)** HONE1, shLacZ and shCOX-2 cells were pre-treated with 50 ng/ml EGF and 10 μM PGE_2_ in serum-free medium for 3 h. Cells were then labeled with DiI and cultured with endothelial cells for 3 h. Cell attachment was examined using a microscope (upper panel). The number of attached cells was determined using three randomly chosen fields under a microscope from three independent experiments (lower panel). **(C)** HONE1 and shFN cells were pre-treated with 50 ng/ml EGF and 10 μM PGE_2_ in serum-free medium for 3 h. Cell attachment was examined under a microscope (upper panel). The number of attached cells was determined using three randomly chosen fields under a microscope from three independent experiments (lower panel). Values are indicated as the means ± S.E.M. Scale bar represents 200 μm.

We found that EGF concomitantly induced cell migration and invasion and fibronectin expression. In addition, an analysis of fibronectin expression between normal and malignant tissues from HNSCC patients in published datasets [39] revealed higher fibronectin expression levels in malignant versus normal tissues (Supplemental Figure S9C). To clarify whether the induction of fibronectin is important for mediating COX-2-induced cell invasion, the effects of EGF and PGE_2_ on cell invasion were examined in shFN cells. Consistent with reduced actin polymerization with COX-2 knockdown (Figure 1B), disruption of actin polymerization following fibronectin depletion suggested that EGF-mediated cytoskeletal rearrangement was dependent on fibronectin expression (Supplemental Figure S9D). Furthermore, we assessed the effect of fibronectin expression on PGE_2_-induced cell transendothelial invasion. As shown in Figure 6A and Supplemental Figure 10A, depletion of fibronectin abolished PGE_2_-induced cell invasion. These results suggest that EGF-induced cell migration and invasion occur through induction of COX-2 expression and activation of the fibronectin/FAK/Rac1/cdc42 signaling pathway.

Distant metastasis relies on tumor cell attachment to blood vessels [44]. Thus, we further tested the possibility that the metastatic process enhanced by EGF-induced COX-2 might occur via regulation of the interaction between tumor and endothelial cells. As shown in Figure 6B, EGF promoted the binding of HONE1 cells to HMEC-1 cells, and this binding was dramatically reduced in shCOX-2 cells. The inhibition of this tumor-endothelial cell interaction in shCOX-2 cells was reversed when cells were treated with both EGF and PGE_2_ (Figure 6B). To further confirm that the activation of COX-2 is required for the tumor-endothelial cell interaction, HNSCC cells were treated with PGE_2_, which dramatically induced binding to endothelial cells, as shown in Figure 6C and Supplemental Figure S10B. In addition, the induction of the tumor-endothelial cell interaction was also blocked in shFN cells (Figure 6C and Supplemental Figure S10B). These results indicate that COX-2 and fibronectin stimulate the binding of tumor cells to endothelial cells, which may result in enhanced capacity to penetrate blood vessels.

### PGE_2_-induced fibronectin expression promotes tumor invasion *in vivo*

**Figure 7:**
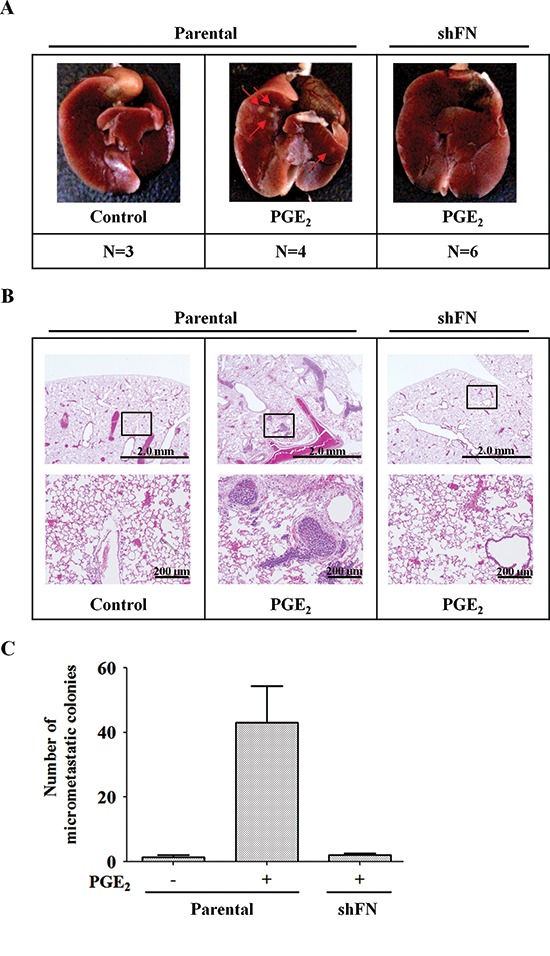
PGE_2_-primed HNSCC metastasis is inhibited in fibronectin knockdown cells **(A)** HONE1 and shFN cells (1 × 10^6^) were treated with 10 μM PGE_2_ in serum-free medium for 3 h and then injected into the tail vein of SCID mice. Arrows point to metastatic nodules. N, number of SCID mice. **(B, C)** Colonies in the lungs were quantified at 2 months, and the average is presented. Quantitation of the number of H&E stained lung nodules from the SCID mice was performed under a microscope. A magnified view of the respective boxed area is shown below each image. Values represent means ± S.E.M.

To determine the effect of fibronectin on PGE_2_-induced metastasis *in vivo*, we investigated the distant dissemination (e.g., pulmonary colonization) of tumor cells using tail vein injection in an animal model. Briefly, parental and shFN cells were pretreated with PGE_2_ for 3 h and then injected into the tail vein of mice. No obvious lung nodules were detected when fibronectin was depleted in the HNSCC cells, whereas the PGE_2_-treated parental tumor cells developed significant nodules (Figure 7A and Supplemental Figure S11A). Hematoxylin and eosin (H&E) staining revealed that the lungs of mice receiving the PGE_2_-treated parental tumor cells contained significantly more and larger micrometastatic colonies than those receiving the shFN cells (Figures 7B and 7C, and Supplemental Figure S11B). These results suggest that fibronectin is essential for PGE_2_- primed HNSCC metastasis.

## DISCUSSION

In the current treatment of head and neck cancers, cetuximab plus radiation therapy improves overall survival in patients without distal metastases [10]. However, few patients benefit from cetuximab, which is the only EGFR-targeted therapy approved for HNSCC [9, 10]. Many studies have indicated that in most patients who respond to EGFR inhibitors, tumors will subsequently become refractory [45]. These studies indicate that the dysregulation of EGFR activation and downstream signaling pathways may interfere with therapeutic efficacy in HNSCC. For example, resistance to cetuximab has been correlated with the dysregulation of EGFR family members HER2 and HER3 [45, 46]. The major impediments to curing advanced HNSCC are tightly related to metastasis, which may contribute to drug resistance. However, the EGFR target that enhances HNSCC metastasis remains unclear. In addressing this challenge, we investigated whether the induction of COX-2 regulates EGF-induced metastasis. Indeed, we found that the autocrine production of PGE_2_ by EGF-induced COX-2 regulates fibronectin expression, which promotes HNSCC metastasis. Our survey of COX-2 and fibronectin expression in clinical human HNSCC samples from a microarray database suggested that up-regulation of COX-2 and fibronectin is associated with malignant HNSCC. Consistent with these results, Pan J et al. revealed that the concomitant expression of EGFR and COX-2 in HNSCC correlated with poor prognosis [47]. Therefore, therapeutically targeting COX-2 and fibronectin in EGFR-stimulated HNSCC metastasis would be of interest because metastasis represents the main clinical challenge after primary tumor resection.

In this study, we first clarified the mechanism by which EGF-induced COX-2 expression promotes HNSCC metastasis. Our results revealed that PGE_2_, the COX-2 product of arachidonic acid, enhances the expression of fibronectin, resulting in enhanced tumor metastasis. COX-2 expression is regulated by inflammatory cytokines and growth factors, which are known to drive COX-2 gene expression through NF-κB and AP-1 activation, respectively [19, 48]. In addition, several reports have demonstrated that fibronectin overexpression enhances COX-2 expression. For example, activation of the α5β1/FAK/p38 MAPK pathway was shown to be involved in fibronectin-induced COX-2 expression in HUVECs, and COX-2 contributed to the angiogenic effect of fibronectin [30]. Fibronectin up-regulates COX-2 expression and PGE_2_ production to enhance MMP-2 activity in rhabdomyosarcoma and promote lung carcinoma cell proliferation [25, 28]. Interestingly, engulfment and cell motility 1 (ELMO1) interacts with COX-2 to further regulate COX-2 activity and increase fibronectin expression to promote the development of renal glomerular disease [49]. In our study, although knockdown of COX-2 dramatically inhibited EGF-induced fibronectin expression, we found that knockdown of fibronectin had no effect on COX-2 expression in EGF-treated HNSCC cells. In addition, fibronectin overexpression enhanced the activation of Rac1/cdc42 but had no effect on COX-2 expression. Interestingly, PGE_2_ alone promoted HNSCC transendothelial invasion through the induction of fibronectin. These results are in contrast to previous reports showing that COX-2 is induced by fibronectin in lung carcinoma, rhabdomyosarcoma and HUVECs [25, 28, 30]. The difference in signaling molecules participating in the EGFR-COX-2-fibronectin signaling pathway may be due to a cell type-specific response to EGF treatment. For example, we observed EGF-induced COX-2 expression in HNSCC cell lines but not in breast cancer, colorectal cancer or lung cancer cell lines. Thus, the EGFR-COX-2-fibronectin pathway is one of various routes to drive EGF mediated HNSCC metastasis. Indeed, EGF-activated FAK and Rac1/cdc42 were decreased in fibronectin-deficient cells, even though COX-2 remained induced by EGF, or in cells pretreated with PGE_2_. These results reveal that COX-2 loses its ability to activate FAK and Rac1/cdc42 when fibronectin is depleted. Taken together, these results indicate that the regulation of fibronectin by COX-2 plays an important role in tumor metastasis.

The induction of MMPs is a major cause of tumor invasion. Several reports have shown that COX-2 inhibitors reduce HNSCC viability and invasion by down-regulating MMP-2, MMP-9 and VEGF secretion [50, 51]. In agreement with these findings, we also found that EGF-induced COX-2 enhanced HNSCC invasion through the induction of MMPs, such as MMP-1, MMP-2, MMP-3 and MMP-9. However, the mechanism involved in COX-2-mediated MMPs expression remains unclear. The induction of MMP-9 by PGE_2_ in TNF-α treated cholangiocarcinoma cells has been reported to occur through the activation of EP2/4 receptors [52]. Therefore, it will be of interest to clarify whether the downstream signaling of EP receptors is essential for EGF-induced MMPs expression. In addition to EGF, IL-1β and TNF-α also trigger MMP-1 and MMP-3 production in gingival fibroblasts. However, except for IL-1β and TNF-α, EGF-induced MMP-1/3 expression was not altered when the cells were treated with a COX-2 inhibitor [53]. The discrepancy between these results and our findings could be due to differences in downstream signaling activated by EGF in normal versus tumor tissues. We found that COX-2 knockdown inhibited EGF-induced MMP-1, MMP-2, MMP-3 and MMP-9 expression and that this phenomenon was reversed by treating the cells with both EGF and PGE_2_. However, except for MMP-2, PGE_2_ alone did not induce the expression of MMP-1, MMP-3 and MMP-9. These results reveal that cooperation with EGF-activated unknown factors is essential for PGE_2_ in the regulation of MMPs expression in HNSCC.

During tumor metastasis, a major step required for successful distant metastasis involves the ability of circulating tumor cells to penetrate blood vessels [54]. We also provide evidence demonstrating that EGF activation induced the COX-2-primed tumor cell metastatic seeding of the lungs. Using tail vein injection in an animal model, EGF-primed tumor cell metastatic seeding of the lungs was significantly inhibited with the depletion of COX-2 expression. Importantly, PGE_2_ also enhanced the formation of lung nodules, and this priming of metastasis was dramatically inhibited in shFN cells. In agreement with our results, TGFβ induces ANGPTL4 expression to prime the attachment of tumor cells to microvessels, resulting in metastatic lung colonization [55]. These studies suggest that the extravasation process, which is primed by growth factor-induced proteins such as COX-2, fibronectin and ANGPTL4, is a limiting step in distal tumor dissemination. In addition, we also found that induction of COX-2 and fibronectin by EGF enhanced the interaction between tumor cells and endothelial cells. Several mediators of pulmonary extravasation have recently been identified, which are up-regulated in the primary tumors of breast cancer patients with lung metastasis [54, 56]. These include epiregulin, COX-2, MMP-1 and MMP-2, proteins that support not only vascular remodeling in primary tumors but also lung extravasation [56]. In conclusion, we speculate that EGF-induced expression and activity of COX-2 and fibronectin promotes tumor metastasis by modulating adhesion between tumor and endothelial cells. These results indicate that disruption of the adhesion signaling axes between endothelial cells and tumor cells may serve to prevent the dissemination of metastatic HNSCC. Therefore, it is important to further elucidate the role of COX-2 and the mechanisms involved in the regulation of EGF-primed HNSCC metastasis.

Although few patients benefit from cetuximab, which is the only EGFR-targeted drug approved for the treatment of HNSCC, because not all cases of HNSCC are dependent on EGFR [1], cetuximab in combination with radiation therapy improves overall survival of HNSCC patients. However, the majority of patients treated with anti-EGFR drugs who have metastasis will suffer from recurrences. Thus, there is no effective therapy for the prevention of metastasis. Here, we provide evidence to indicate that the inhibition of COX-2 efficiently prevented lung metastasis of HNSCC. In addition, significantly increased COX-2 expression was observed in HNSCC [57]. Therefore, a combination therapy involving anti-EGFR and COX-2 inhibitors is a possible chemotherapeutic approach for treatment and metastasis prevention in HNSCC patients. Indeed, efficacy studies of dual EGFR/COX-2 inhibition are justified [58]. In conclusion, we demonstrate the clinical and biological function of EGF-induced COX-2 in HNSCC, providing evidence that COX-2 has an important role in HNSCC metastasis. COX-2 and fibronectin levels were increased upon the activation of EGFR signaling, leading to tumor metastasis. Measurement of COX-2 and fibronectin levels in HNSCC may provide clinically useful prognostic biomarkers in HNSCC metastasis and combinatorial inhibition of both COX-2 and fibronectin might provide a novel therapeutic approach for EGFR-overexpressing HNSCC.

## MATERIALS AND METHODS

### Cell culture

The FaDu head and neck cancer cell line was purchased from American Type Culture Collection (ATCC, Manassas, VA, USA). The human microvascular endothelial cell line (HMEC-1) was kindly provided by Dr. Trai-Ming Yeh (Department of Medical Laboratory Science and Biotechnology, Medical College, National Cheng Kung University). The epidermal carcinoma (A431), cervical cancer (HeLa), lung carcinoma (A549), oral squamous cell carcinoma (SCC4, SCC25), breast cancer (MDA-MB-231, MDA-MB-468), and colon cancer (SW480 and SW620) cell lines were purchased from ATCC (Manassas, VA, USA). The head and neck cancer cell lines HONE1, TU183, UMSCC1 and OEC-M1 were kindly provided by Dr. Kwang-Yu Chang (National Health Research Institutes, Taiwan) [59]. The TU183, UMSCC1, A431, HeLa and A549 cell lines were maintained at 37°C under 5% CO_2_ in 10-cm plastic dishes containing 10 ml of Dulbecco's modified eagle's medium (Invitrogen, Grand Island, NY, USA) supplemented with 10% fetal bovine serum (Invitrogen), 100 μg/ml streptomycin (Invitrogen), and 100 units/ml penicillin (Invitrogen). The HONE1 and OEC-M1 cell lines were maintained at 37°C under 5% CO_2_ in 10-cm plastic dishes containing 10 ml of RPMI 1640 medium (Invitrogen) supplemented with 10% fetal bovine serum, 100 μg/ml streptomycin, and 100 units/ml penicillin. The FaDu cell line was maintained at 37°C under 5% CO_2_ in 10-cm plastic dishes containing 10 ml of Eagle's minimum essential medium (Invitrogen) supplemented with 10% fetal bovine serum, 100 μg/ml streptomycin, and 100 units/ml penicillin. The MDA-MB-231, MDA-MB-468, SW480 and SW620 cell lines were maintained at 37°C under 5% CO_2_ in 10-cm plastic dishes containing 10 ml of Leibovitz's L-15 medium (Invitrogen) supplemented with 10% fetal bovine serum, 100 μg/ml streptomycin, and 100 units/ml penicillin. The SCC4 and SCC25 cell lines were maintained at 37°C under 5% CO_2_ in 10-cm plastic dishes containing 10 ml of DMEM:F12 medium (Invitrogen) supplemented with 10% fetal bovine serum, 100 μg/ml streptomycin, and 100 units/ml penicillin. The HMEC-1 cells was maintained in MCDB131 culture medium (Sigma-Aldrich, St Louis, MO, USA) supplemented with 10% fetal bovine serum, 100 μg/ml streptomycin, 100 units/ml penicillin, and 15 μg/ml endothelial cell growth supplement (ECGS) (Millipore, Bedford, MA, USA).

### Immunofluorescence

Cells were seeded onto glass slides overnight and fixed with 4% paraformaldehyde (Sigma-Aldrich) in phosphate-buffered saline at 4°C for 10 min. The cells were then rinsed with phosphate-buffered saline three times and permeabilized with 1% Triton X-100 for 7 min. Next, the cells were pretreated with 1% bovine serum albumin in phosphate buffered saline at 25°C for 60 min and incubated with phalloidin FITC (Sigma-Aldrich) at a dilution of 1:500 for 1 h. Finally, the cells were washed with phosphate-buffered saline, mounted in 90% glycerol containing 4′,6-diamidino-2-phenylindole (DAPI) (Invitrogen), and examined using a microscope (model DMI 4000 B; Leica, Wetzlar, Germany).

### Anchorage-independent soft agar growth assay

Briefly, 1.5 ml per well of base agar matrix was added to a 6-well plate. After solidification, 5000 cells were plated in an agar matrix layer on top of the base. Wells were topped with 1 ml of complete medium and incubated for 14 days at 37°C with 5% CO_2_ to form colonies, which were then stained with crystal violet (0.4 g/l; Sigma-Aldrich).

### Migration and invasion assays

Both assays were performed using Millicell™ hanging cell culture inserts (polyethylene terephthalate (PET) membranes with 8 μm pores) (Millipore). For the transwell migration assay, 2 × 10^5^ cells were plated in serum-free medium containing 50 ng/ml EGF (Invitrogen) or 10 μM PGE_2_ (Sigma-Aldrich) and placed in the upper chamber for 15 h, while the lower chamber was filled with serum-free medium with or without 50 ng/ml EGF. The cells in the upper chamber were removed and the migrated cells at the bottom of the PET membrane were fixed with 4% paraformaldehyde and stained with 0.1% crystal violet. For the invasion assay, 2 × 10^5^ cells were plated in serum-free medium containing 50 ng/ml EGF or 10 μM PGE_2_ and placed in the upper chamber on a 10% Matrigel-coated membrane, while the lower chamber was filled with serum-free medium. After incubation for 48 h, the cells in the upper chamber were removed and the invaded cells at the bottom of the PET membrane were fixed with 4% paraformaldehyde and stained with 0.1% crystal violet. In both assays, the number of invading cells was determined in three randomly chosen fields under the microscope for three independent experiments.

### Transendothelial invasion assay

The invasion assay was performed using Millicell™ hanging cell culture inserts (polyethylene terephthalate (PET) membranes with 8 μm pores) (Millipore). HMEC-1 cells (1 × 10^5^ cells per well) were plated on the upper chamber and allowed to grow to confluence, and then 10% Matrigel was loaded into the chamber. Tumor cells were treated with 50 ng/ml EGF or 10 μM PGE_2_ in serum-free medium and then stained with 1,1′-dioctadecyl-3,3,3′,3′-tetramethyl-indocarbocyanine perchlorate (DiI) (Invitrogen) for 30 min. DiI-stained tumor cells (2 × 10^5^) were then loaded into the chamber, which was filled with serum-free medium, and incubated for 2 days. Cells on the apical side of each insert were scraped off. Invasion to the basolateral side of the membrane was visualized using an immunofluorescent microscope. The number of invading cells was determined in three randomly chosen fields under the microscope for three independent experiments.

### Western blotting

Analytical 12% SDS-PAGE was performed, and 30 μg of protein were analyzed for each condition, unless otherwise stated. For immunoblotting, proteins in the SDS gels were transferred to a polyvinylidene difluoride membrane using an electroblot apparatus. Antibodies against human fibronectin (Santa Cruz Biotechnology, Inc., Santa Cruz, CA), FAK (Cell Signaling Technology, Danvers, MA), p-FAK (Epitomics, Burlingame, CA, USA), N-cadherin (Epitomics, Burlingame, CA, USA), E-cadherin (Epitomics, Burlingame, CA, USA), Snail1 (bs-1371R; Bioss, Boston, MA, USA), COX-2 (Lab Vision Corp., Fremont, CA), c-Jun (Santa Cruz Biotechnology), AKT and p-AKT (both from Cell Signaling Technology, Danvers, MA), p-Rac1/cdc42 (Cell Signaling Technology), and α-tubulin and β-actin (both from Sigma-Aldrich) were used as the primary antibodies. Mouse or rabbit IgG antibodies coupled to horseradish peroxidase were used as secondary antibodies. An enhanced chemiluminescence kit (Supersignal West Pico Chemiluminescence kit; Pierce, Rockford, IL) was used for detection. The FAK inhibitor Y15 was purchased from Sigma-Aldrich (St. Louis, MO).

### Reverse transcription–polymerase chain reaction

Total RNA was isolated using the TRIzol RNA extraction kit (Invitrogen), and 2 μg of RNA was subjected to reverse transcription–polymerase chain reaction (PCR) with SuperScript^TM^II (Invitrogen). The following primers were used: MMP-1 (sense, 5′-ATGCACAGCTTTCCTCCACT-3′; antisense 5′-TTCCCAGTCACTTTCAGCCC -3′), MMP-2 (sense, 5′-GCAAGTTTCCATTCCGC-3′; antisense 5′-GTCGTCATCGTAGTTGGC-3′), MMP-3 (sense, 5′-GCAAGACAGCAAGGCATAGAG-3′; antisense 5′- CCGTCACCTCCAATCCAAGG-3′), MMP-7 (sense, 5′- GCTACAGTGGGAACAGGCTC-3′; antisense 5′-TGGCCCATCAAATGGGTAGG-3′), MMP-9 (sense, 5′-ACCTCGAACTTTGACAGCGACA-3′; antisense 5′-GATGCCATTCACGTCGTCCTTA-3′), MMP-13 (sense, 5′-AACATCCAAAAACGCCAGAC-3′; antisense 5′-GGAAGTTCTGGCCAAAATGA-3′), Slug (sense, 5′- GAGAGCTGCAAGAGCATGGA-3′, antisense, 5′-GGCAACCAGACAACCGACAT-3′), Twist (sense, 5′-GCCGGAGACCTAGATGTCATTG-3′, antisense, 5′- AGTGGCTGATT GGCACGAC-3′), Vimentin (sense, 5′-TGGCCGACGCCATCAACACC-3′, antisense, 5′- CACCTCGACGCGGGCTTTGT-3′), N-cadherin (sense, 5′-GTGCCATTAGCCAAGGGAATTCAGC-3′, antisense, 5′-GCGTTCCTGTTCCA CTCATAGGAGG-3′), and GAPDH (sense, 5′-CCATCACCATCTTCCAGG AG-3′, antisense, 5′-CCTGCTTCACCACCTTCTTG-3′). The PCR products were separated by 1% agarose gel electrophoresis and visualized with ethidium bromide staining.

### Knockdown experiments

The hairpins targeting COX-2 (shCOX-2) and fibronectin 1 (shFN) and a non-targeting hairpin (shLacZ) were obtained from the RNAi Core of the Research Center of Clinical Medicine, National Cheng Kung University Hospital in the pLKO.1 lentiviral backbone. Cells were selected in 2 μg/ml puromycin for 3 days and then expanded for 1–2 weeks before analysis. shCOX-2 #1 and shCOX-2 #2 stable cell lines were selected from the same target sequence. Hairpin TRC clone IDs and target sequences were as follows:

shLacZ / TRCN0000072223, TGTTCGCAT TATCCGAACCAT

shCOX-2 / TRCN0000045533, GCTGAATTTA ACACCCTCTAT

shFN / TRCN0000064828, CGTGGTTGTA TCAGGACTTAT.

### Transfection with siRNA oligonucleotides

Transient transfection of cells with 20 nM prostaglandin-endoperoxide synthase 2 (PTGS2) (siRNA IDs: HSS183839, HSS183840) or fibronectin (FN) (siRNA IDs: HSS103780, HSS103782) siRNA oligonucleotides were performed using RNAiMAX (Invitrogen, Grand Island, NY) according to the manufacturer's instructions with slight modifications. For use in transfection, 1.5 μl of RNAiMAX was incubated with COX-2 siRNA, fibronectin siRNA or scramble siRNA (Invitrogen, Grand Island, NY) in 1.5 ml of Opti-MEM medium for 30 min at room temperature. Following the change of Opti-MEM medium to 3 ml of fresh culture medium, cells were incubated for an additional 24 hours, unless stated otherwise.

### Plasmid transfection and luciferase assays

Luciferase vectors containing the MMP-1, MMP-3, MMP-7, MMP-9, MMP-10 and COX-2 gene promoters were used. Transient transfection of cells with plasmids was performed with Lipofectamine 2000 (Invitrogen) according to the manufacturer's instructions but with slight modification. The luciferase activity in cell lysate was determined as described previously [19].

### Cell adhesion assay

Briefly, HONE1 or FaDu cells were treated with 50 ng/ml EGF or 10 μM PGE_2_ in serum-free medium for 3 h then labeled for 30 min at 37°C with DiI (Invitrogen) and washed twice with phosphate-buffered saline. The medium was removed from the wells, and HONE1 or FaDu cells (1.5 × 10^5^ cells/ml serum-free medium) were added to a monolayer of HMEC-1 cells. After incubation for 30 min at 37°C, the wells were gently washed twice with phosphate-buffered saline to remove non-adherent cells. The cells were photographed and quantified under a fluorescence microscope.

### Tumor metastasis assay in an animal model

Tumor metastasis was determined by tail vein intravenous injection of cancer cells into 4- to 6-week-old male severe combined immunodeficiency (SCID) mice. Briefly, each animal was injected with 1×10^6^ cells mixed with phosphate-buffered saline, and all mice were sacrificed up to 2 months after injection. All mice were obtained from the National Cheng Kung University Laboratory Animal Center (Tainan, Taiwan) and the National Laboratory Animal Center (Tainan, Taiwan). All animal experiments in this study were approved by the Laboratory Animal Committee of National Cheng Kung University. H&E staining was performed by the Human Biobank, Research Center of Clinical Medicine, National Cheng Kung University Hospital.

### Statistical analysis

Data were expressed as means ± S.E.M. Statistical analysis was performed using GraphPad Prism 5 statistical software (La Jolla, CA, USA) for Microsoft Windows.

## SUPPLEMENTARY FIGURES


