# Supplementary material for: Epidermal growth factor-induced cyclooxygenase-2 enhances head and neck squamous cell carcinoma metastasis through fibronectin up-regulation
Source: Oncotarget. 2014 Dec 22;6(3):1723–39. doi: 10.18632/oncotarget.2783 (PMC4359327; doi:10.18632/oncotarget.2783)
Supplement: Supplementary file 1 [file oncotarget-06-1723-s001.pdf]

SUPPLEMENTARY FIGURES

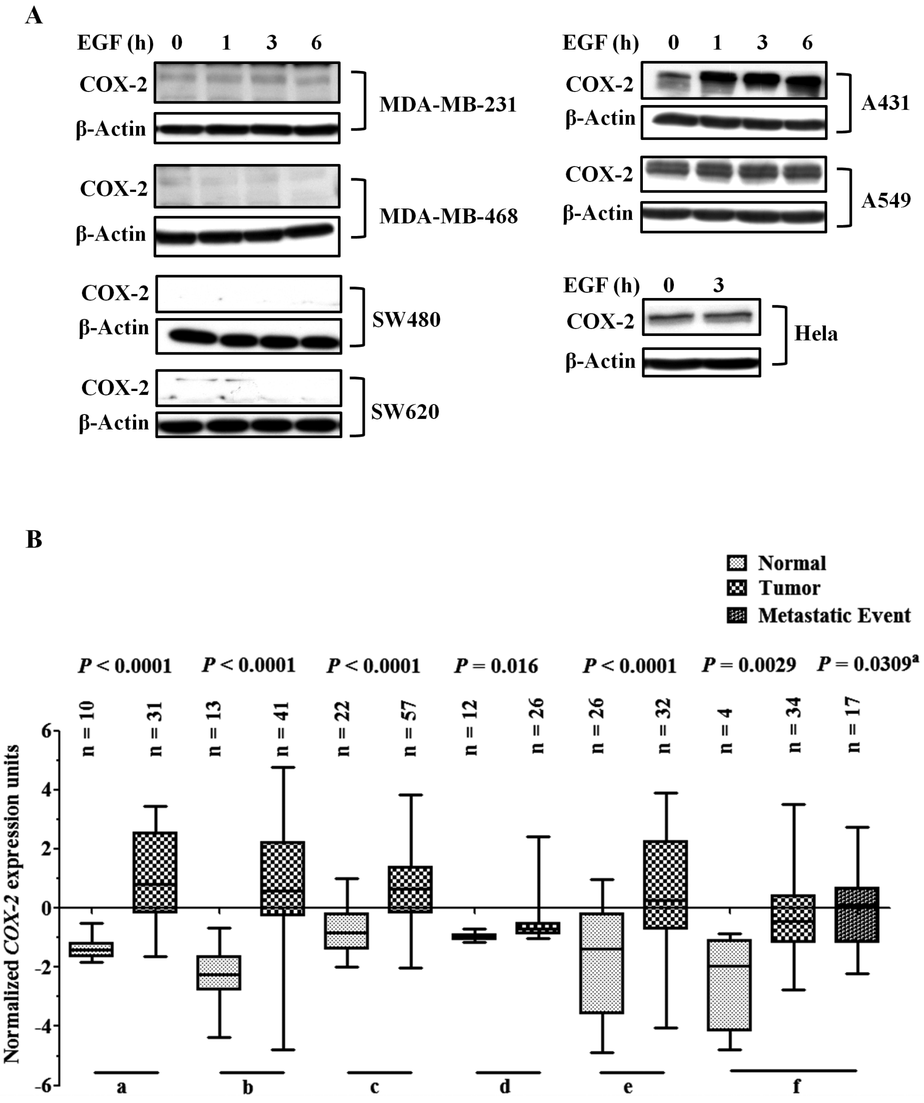

Supplementary Figure S1: COX-2 expression in EGF-treated tumor cell lines and tumor tissues from human HNSCC patients. **(A)** Cells were treated with 50 ng/ml EGF in serum-free medium for the indicated period of time. Cell lysates were prepared, subjected to SDS-PAGE and analyzed by western blotting with antibodies against COX-2 and β-actin. **(B)** Oncomine boxed plot of *COX-2* expression levels between human normal and malignant tissues or metastatic event from HNSCC patients in multiple datasets from ref. 1 (a), ref. 2 (b), ref. 3 (c), ref. 4 (d), ref. 5 (e) and ref. 6 (f). 1. Sengupta S, et al. (2006) Genome-wide expression profiling reveals EBV-associated inhibition of MHC class I expression in nasopharyngeal carcinoma. *Cancer Res*. 66:7999–8006. 2. Ginos MA, et al. (2004) Identification of a gene expression signature associated with recurrent disease in squamous cell carcinoma of the head and neck. *Cancer Res*. 64:55–63. 3. Peng CH, et al. (2011) A novel molecular signature identified by systems genetics approach predicts prognosis in oral squamous cell carcinoma. *PLoS One*. 6:e23452. 4. Ye H, et al. (2008) Transcriptomic dissection of tongue squamous cell carcinoma. B*MC Genomics*. 9:69. 5. Estilo CL, et al. (2009) Oral tongue cancer gene expression profiling: Identification of novel potential prognosticators by oligonucleotide microarray analysis. *BMC Cancer*. 9:11. 6. Cromer A, et al. (2004) Identification of genes associated with tumorigenesis and metastatic potential of hypopharyngeal cancer by microarray analysis. *Oncogene*. 23:2484–98. [a], metastatic event versus normal.

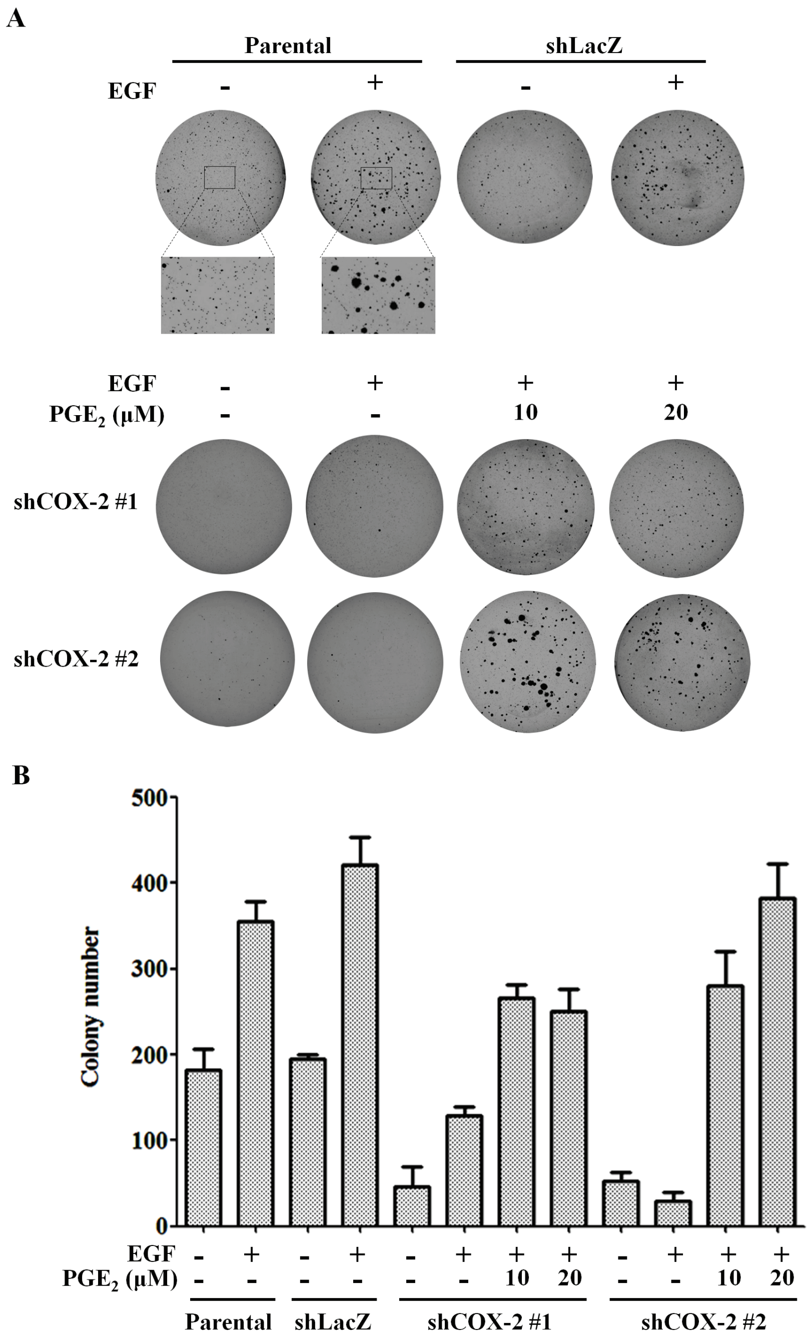

Supplementary Figure S2: Induction of COX-2 is essential for EGF-induced anchorage-independent growth of HNSCC cells. **(A)** Tumor transformation was examined using the soft agar transformation assay as describe in the "Materials and methods". HONE1 and shCOX-2 cells were treated with 50 ng/ml EGF and PGE_2. After 14 days, images were captured using a microscope. Magnified views of the boxed areas are shown below the images. **(B)** The number of colonies was calculated from three independent experiments. Values are means ± S.E.M. of three determinations.

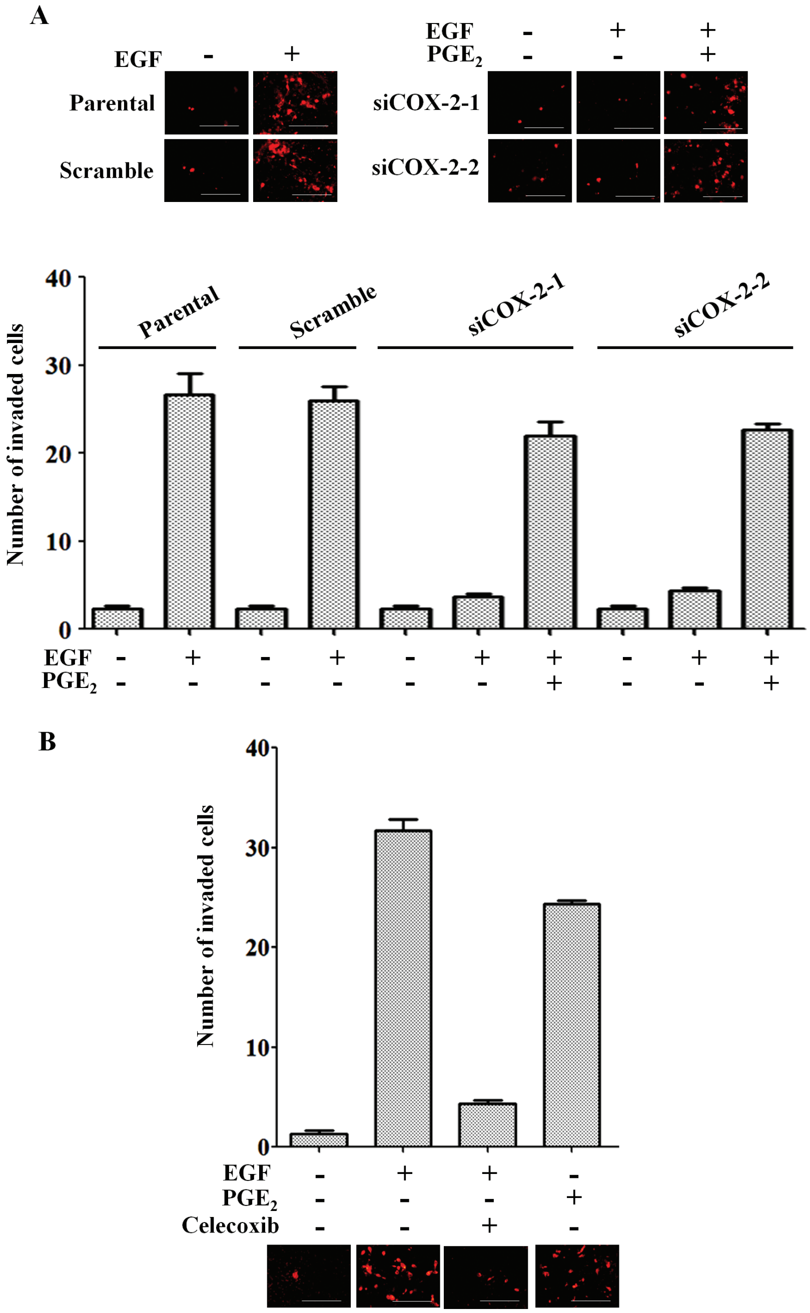

Supplementary Figure S3: EGF-enhanced HNSCC cell invasion is inhibited by COX-2 depletion. **(A)** The invasive properties of HONE1 and siCOX-2 cells were analyzed using the transendothelial invasion assay. Cells were transfected with 2 different COX-2 siRNA oligonucleotides (siCOX-2) or control siRNA (scramble) for 24 h before the assays. Cells were treated with 50 ng/ml EGF or 10 µM PGE_2 in serum-free medium for 48 h. Images of invaded cells were captured using a microscope (upper panel). Invaded cells were quantified under a microscope using three randomly chosen fields from three independent experiments (lower panel). Scale bar represents 200 µm. (B) The invasive properties of HONE1 cells were analyzed using the transendothelial invasion assay. Cells were treated with 50 ng/ml EGF, 10 µM PGE_2 and 10 µM celecoxib in serum-free medium for 48 h. The number of invaded cells was determined under a microscope using three randomly chosen fields from three independent experiments (upper panel). Images of invaded cells were captured using a microscope (lower panel). Values are indicated as the means ± S.E.M. Scale bar represents 200 µm.

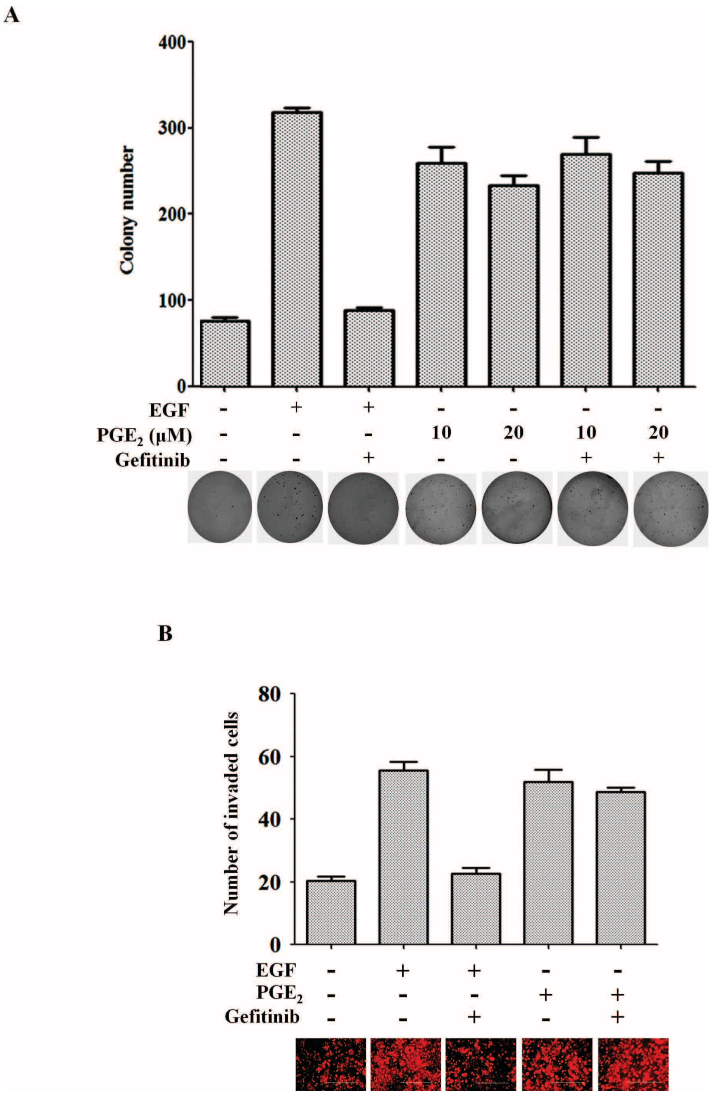

Supplementary Figure S4: The EGFR inhibitor gefitinib reduces EGF- but not PGE_2-induced anchorage-independent growth and invasion. **(A)** Tumor transformation was examined using the soft agar transformation assay as described in the "Material and methods". HONE1 cells were treated with 50 ng/ml EGF, 10 μM PGE_2 and 3 μM gefitinib. After 14 days, images were captured under a microscope. The number of colonies was calculated from three independent experiments. Values are mean ± s.e.m. of three determinations. **(B)** The invasive properties of HONE1 cells were analyzed using the transendothelial invasion assay. Cells were treated with 50 ng/ml EGF, 10 μM PGE_2 and 10 μM gefitinib in serum-free medium for 48 h. The number of invaded cells was determined under a microscope using three randomly chosen fields from three independent experiments (upper panel). Images of invaded cells were captured under a microscope (lower panel). Values represent the means ± S.E.M. Scale bar represents 200 μm.

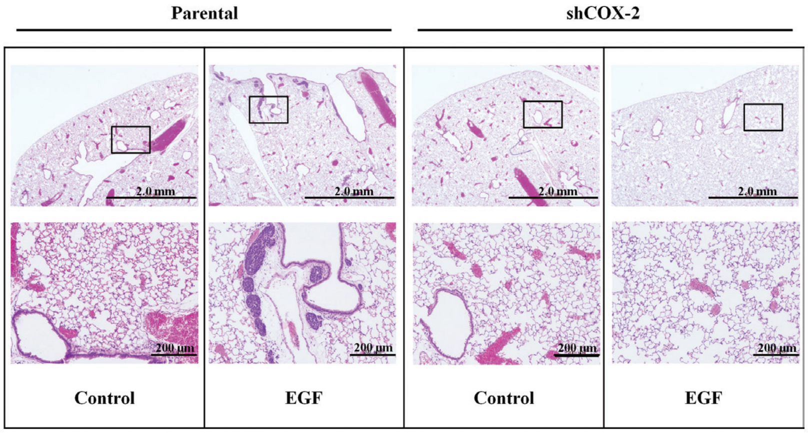

Supplementary Figure S5: EGF-primed HNSCC metastasis is inhibited in COX-2 knockdown cells. HONE1 and shCOX-2 cells (1 x 10^6) were treated with 50 ng/ml EGF in serum-free medium for 3 h and then injected into the tail vein of SCID mice. Colonies in the lungs were examined using H&E staining at 2 months.

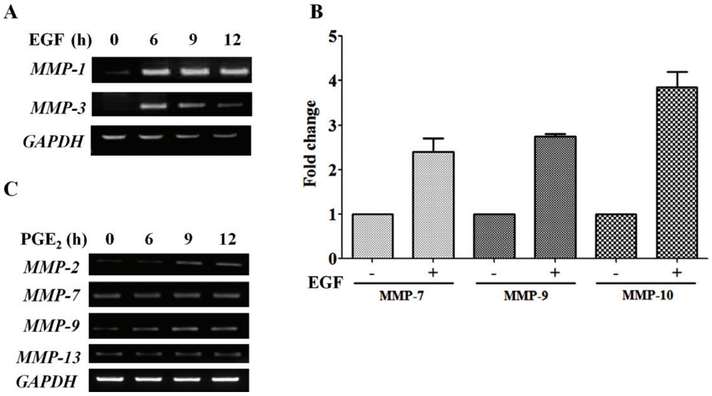

Supplementary Figure S6: MMP expression in EGF and PGE_2-treated cells. HONE1 cells were treated with 50 ng/ml EGF or 10 µM PGE_2 in serum-free medium for the indicated period of time. **(A)** Total RNA was extracted for reverse transcription PCR with *MMP-1, MMP-3* and *GAPDH* primers. **(B)** HONE1 cells were transfected with *MMP-7, MMP-9* and *MMP-10* promoters by lipofection. Cells were treated with 50 ng/ml EGF for 24 h. Luciferase activity and protein concentrations were then determined and normalized. Values represent means ± S.E.M. of three determinations. **(C)** Total RNA was extracted for reverse-transcription PCR with *MMP-2, MMP-7, MMP-9, MMP-13* and *GAPDH* primers.

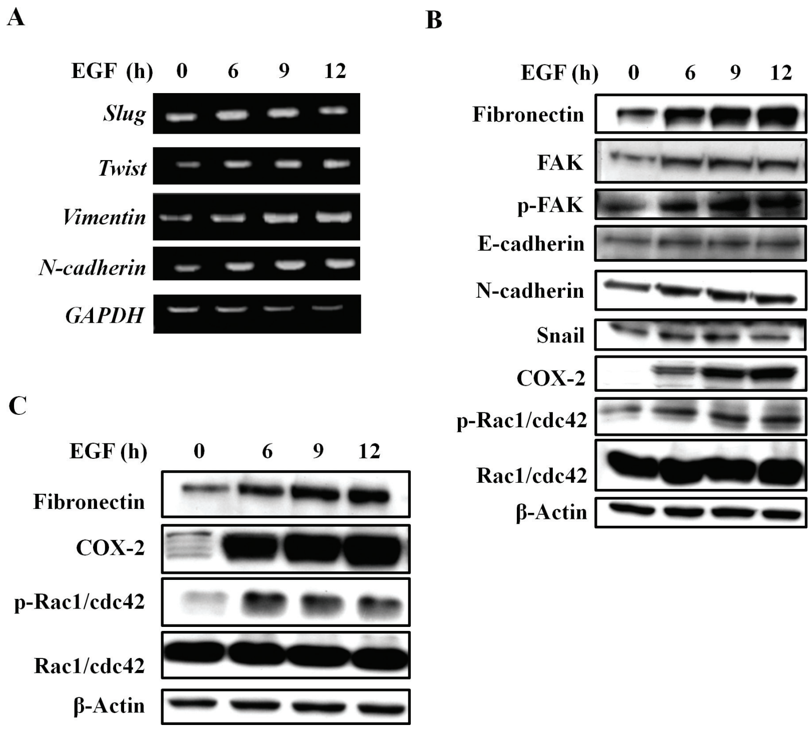

Supplementary Figure S7: Alterations in EMT markers in EGF-treated cells. HONE1 cells were treated with 50 ng/ml EGF in serum-free medium for the indicated period of time. **(A)** Total RNA was extracted for reverse-transcription PCR with *Slug, Twist, Vimentin, N-cadherin* and *GAPDH* primers. **(B)** Lysates of HONE1 cells were prepared, subjected to SDS-PAGE and analyzed by western blotting with antibodies against fibronectin, FAK, N-cadherin, E-cadherin, COX-2, snail, β-actin, Rac1/cdc42 and phosphorylated FAK and Rac1/cdc42. **(C)** Lysates of FaDu cells were prepared, subjected to SDS-PAGE and analyzed by western blotting with antibodies against fibronectin, COX-2, β-actin, Rac1/cdc42 and phosphorylated Rac1/cdc42.

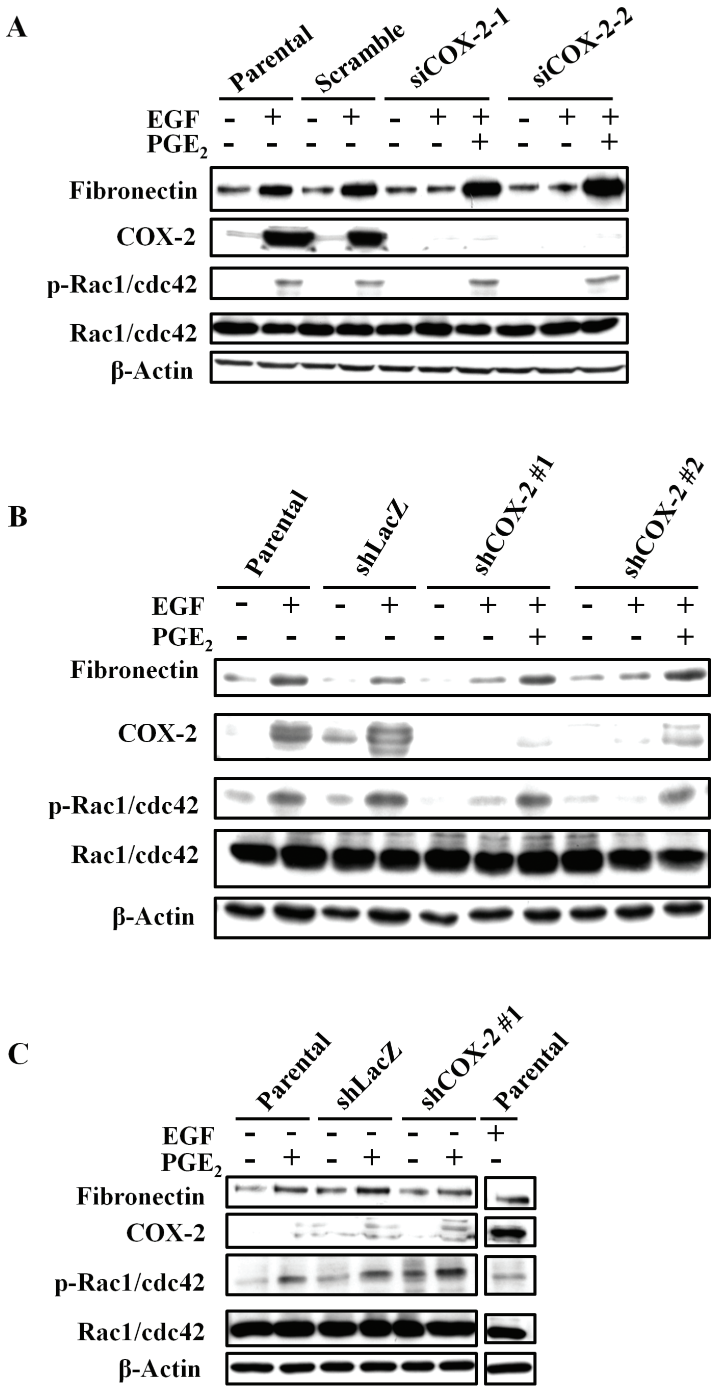

Supplementary Figure S8: The activation of COX-2 is essential for EGF-induced expression of fibronectin and activation of Rac1/cdc42. **(A)** HONE1 cells were transfected with 2 different COX-2 siRNA oligonucleotides (siCOX-2) or control siRNA (scramble) for 24 h before the assays. HONE1 cells were treated with 50 ng/ml EGF and 10 μM PGE_2 in serum-free medium for 9 h. Cell lysates were prepared, subjected to SDS-PAGE and analyzed by western blotting with antibodies against fibronectin, COX-2, Rac1/cdc42, phosphorylated Rac1/cdc42 and β-actin. **(B)** FaDu and shCOX-2 cells were treated with 50 ng/ml EGF and 10 μM PGE_2 in serum-free medium for 9 h. Cell lysates were prepared, subjected to SDS-PAGE and analyzed by western blotting with antibodies against fibronectin, COX-2, Rac1/cdc42, phosphorylated Rac1/cdc42 and β-actin. **(C)** HONE1, shLacZ and shCOX-2 #1 cells were treated with 50 ng/ml EGF and 10 μM PGE_2 in serum-free medium for 9 h. Cell lysates were prepared, subjected to SDS-PAGE and analyzed by western blotting with antibodies against fibronectin, COX-2, Rac1/cdc42, phosphorylation of Rac1/cdc42 and β-actin.

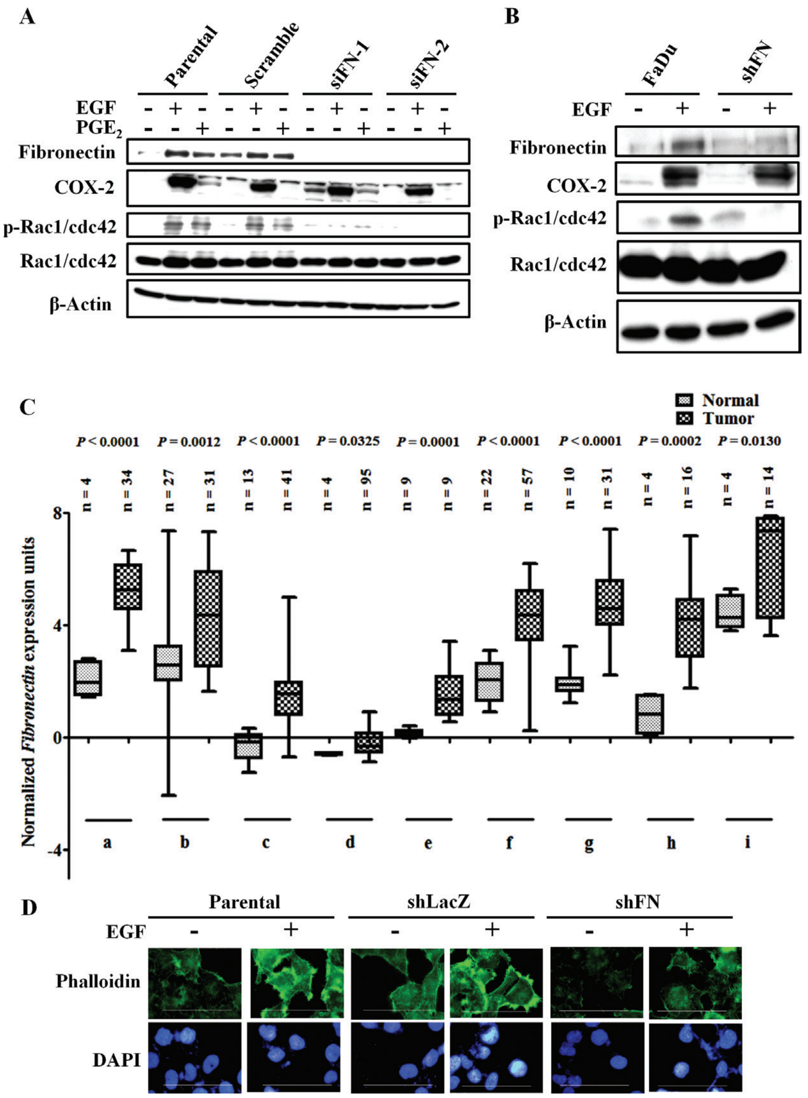

Supplementary Figure S9: Fibronectin expression is increased in HNSCC tumor tissues, and fibronectin is essential for EGF- and PGE_2-induced Rac1/cdc42 activation. **(A)** HONE1 cells were transfected with 2 different fibronectin siRNA oligonucleotides (siFN) or control siRNA (scramble) for 24 h before the assays. HONE1 cells were treated with 50 ng/ml EGF or 10 µM PGE_2 in serum-free medium for 9 h. Cell lysates were prepared, subjected to SDS-PAGE and analyzed by western blotting with antibodies against fibronectin, COX-2, β-actin, Rac1/cdc42 and phosphorylated Rac1/cdc42. **(B)** FaDu and shFN cells were treated with 50 ng/ml EGF in serum-free medium for 9 h. Cell lysates were prepared, subjected to SDS-PAGE and analyzed by western blotting with antibodies against fibronectin, COX-2, β-actin, Rac1/cdc42 and phosphorylated Rac1/cdc42. **(C)** Oncomine boxed plot of *fibronectin* expression levels between human normal and tumor tissues from HNSCC patients in multiple datasets from ref. 1 (a), ref. 2 (b), ref. 3 (c), ref. 4 (d), ref. 5 (e), ref. 6 (f), ref. 7 (g), ref. 8 (h), and ref. 9 (i). 1. Cromer A, et al. (2004) Identification of genes associated with tumorigenesis and metastatic potential of hypopharyngeal cancer by microarray analysis. *Oncogene*. 23:2484–98. 2. Estilo CL, et al. (2009) Oral tongue cancer gene expression profiling: Identification of novel potential prognosticators by oligonucleotide microarray analysis. *BMC Cancer*. 9:11. 3. Ginos MA, et al. (2004) Identification of a gene expression signature associated with recurrent disease in squamous cell carcinoma of the head and neck. *Cancer Res*. 64:55–63. 4. Giordano TJ, et al. (2006) Delineation, functional validation, and bioinformatic evaluation of gene expression in thyroid follicular carcinomas with the PAX8-PPARG translocation. *Clin Cancer Res*. 12:1983–93. 5. He H, et al. (2005) The role of microRNA genes in papillary thyroid carcinoma. *Proc Natl Acad Sci U S A*. 102:19075–80. 6. Peng CH, et al. (2011) A novel molecular signature identified by systems genetics approach predicts prognosis in oral squamous cell carcinoma. *PLoS One*. 6:e23452. 7. Sengupta S, et al. (2006) Genome-wide expression profiling reveals EBV-associated inhibition of MHC class I expression in nasopharyngeal carcinoma. *Cancer Res*. 66:7999–8006. 8. Toruner GA, et al. (2004) Association between gene expression profile and tumor invasion in oral squamous cell carcinoma. *Cancer Genet Cytogenet*. 154:27–35. 9. Vasko V, et al. (2007) Gene expression and functional evidence of epithelial-to-mesenchymal transition in papillary thyroid carcinoma invasion. *Proc Natl Acad Sci U S A*. 104:2803–8. **(D)** HONE1, shLacZ and shFN cells were treated with 50 ng/ml EGF in serum-free medium for 9 h, fixed with 4% paraformaldehyde and labeled with the F-actin-specific fluorescent dye, phalloidin. DNA was stained with DAPI. Immunofluorescence images were captured under a microscope. Scale bar represents 100 µm.

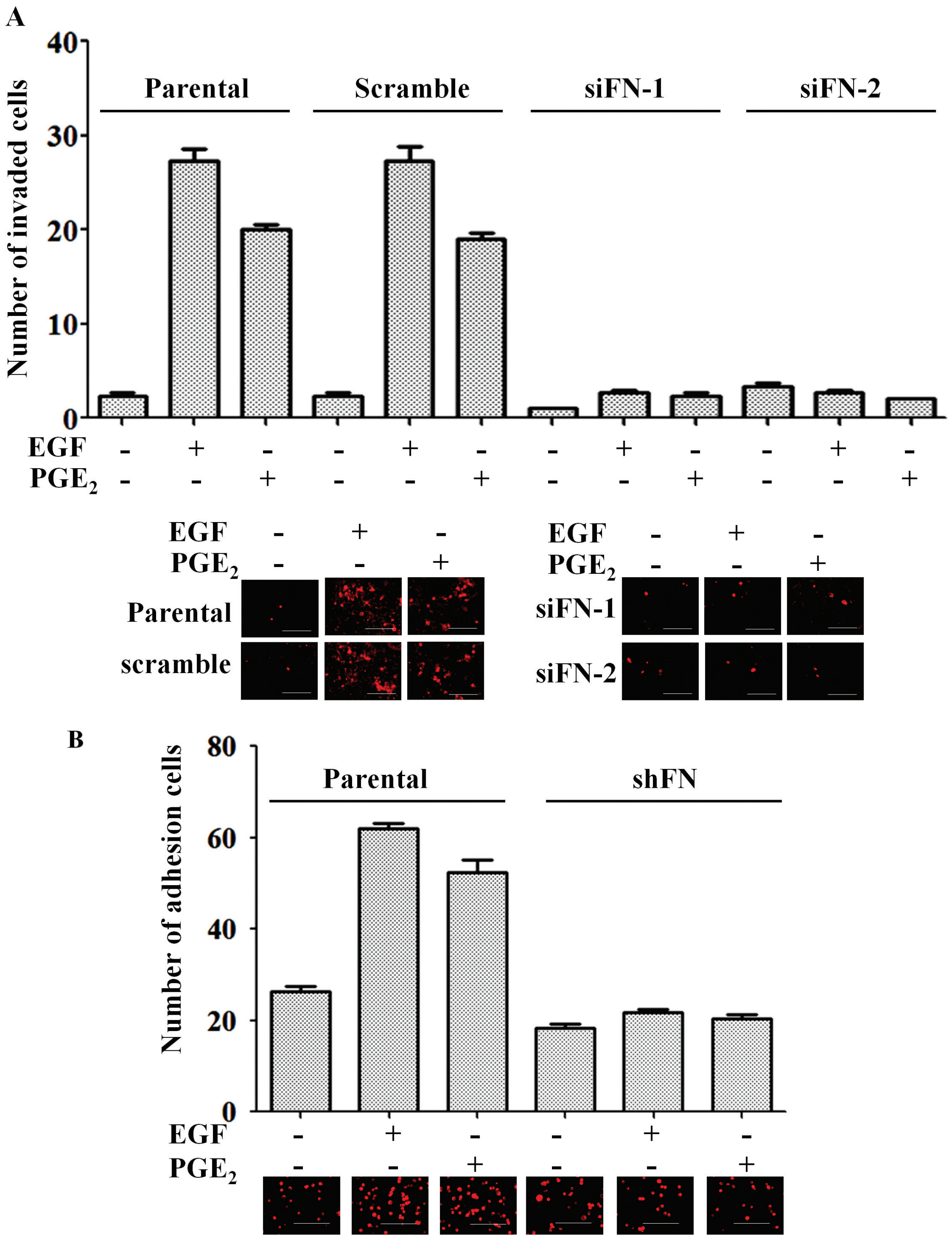

Supplementary Figure S10: Knockdown of fibronectin inhibits EGF- and PGE_2-enhanced HNSCC invasion and adhesion to endothelial cells. (A) Cells were transfected with 2 different fibronectin siRNA oligonucleotides or control siRNA (scramble) for 24 h before the assays. Cells were treated with 50 ng/ml EGF or 10 μM PGE_2 in serum-free medium for 48 h. The number of invaded cells was determined under a microscope using three randomly chosen fields from three independent experiments (upper panel). Images of invaded cells were captured using a microscope (lower panel). Scale bar represents 200 μm. **(B)** FaDu and shFN cells were pre-treated with 50 ng/ml EGF and 10 μM PGE_2 in serum-free medium for 3 h. Cells were then labeled with DiI and cultured with endothelial cells in serum-free medium for 3 h. The attachment of cells was examined using a microscope (lower panel). The number of attached cells was counted under a microscope using three randomly chosen fields from three independent experiments (upper panel). Values represent the means ± S.E.M. Scale bar represents 200 μm.

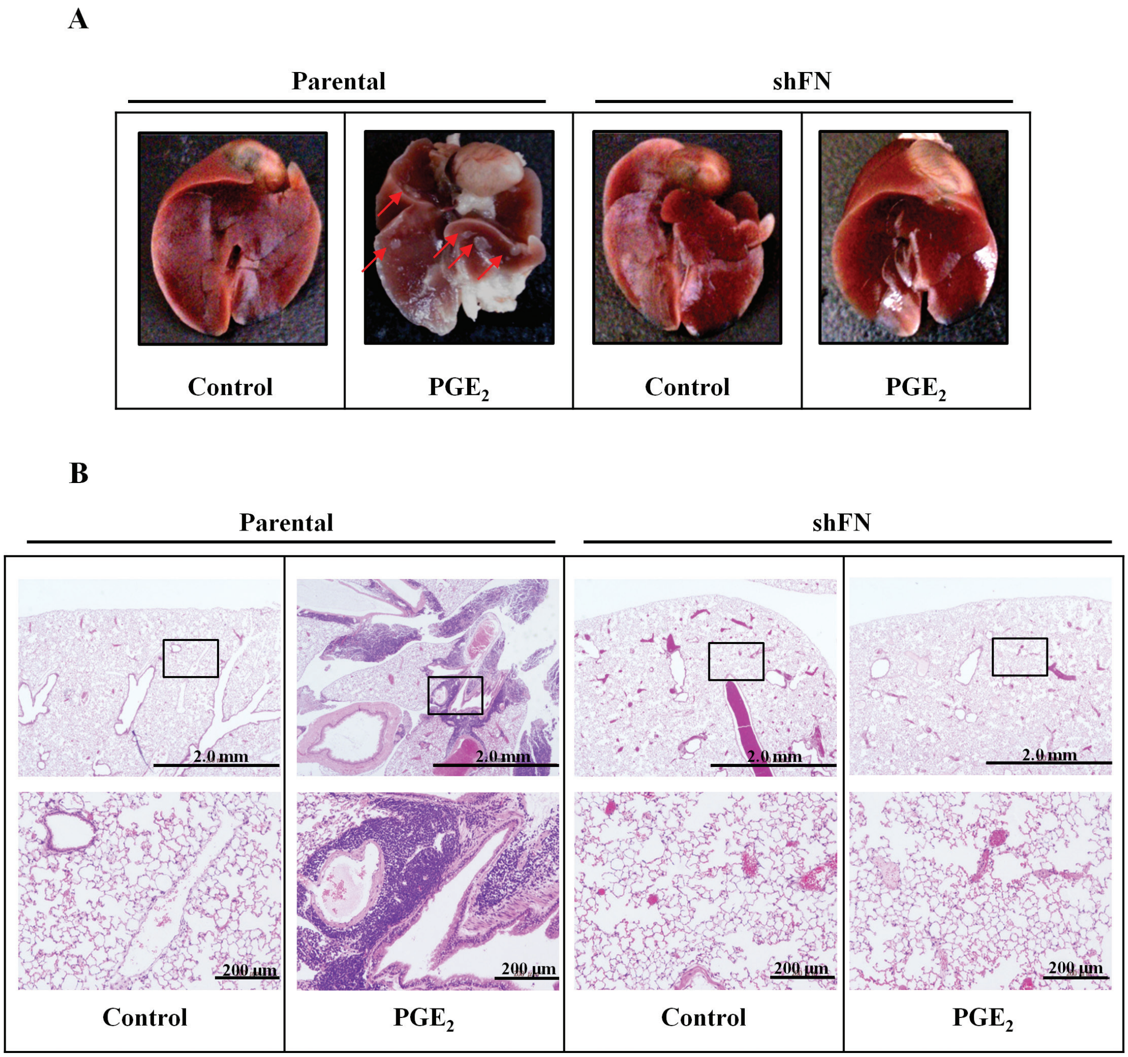

Supplementary Figure S11: PGE$_2$-primed HNSCC metastasis is inhibited in fibronectin knockdown cells. (A) FaDu and shFN cells (1 x 10^6) were treated with 10 µM PGE$_2$ in serum-free medium for 3 h and then injected into the tail vein of SCID mice. Arrows point to metastatic nodules. **(B)** H&E staining of lung colonies at 2 months are shown. A magnified view of the respective boxed area is shown below each image.
